# Cleft Orthodontic Care in Europe: A Cross-Sectional Survey

**DOI:** 10.3390/healthcare10081555

**Published:** 2022-08-17

**Authors:** Inês Francisco, Gregory S. Antonarakis, Francisco Caramelo, Maria Helena Fernandes, Francisco Vale

**Affiliations:** 1 Institute of Orthodontics, Faculty of Medicine, University of Coimbra, 3000-075 Coimbra, Portugal; 2 Coimbra Institute for Clinical and Biomedical Research (iCBR), Area of Environment Genetics and Oncobiology (CIMAGO), Faculty of Medicine, University of Coimbra, 3000-075 Coimbra, Portugal; 3 Division of Orthodontics, University Clinic of Dental Medicine, University of Geneva, 1211 Geneva, Switzerland; 4 Laboratory of Biostatistics and Medical Informatics (LBIM), Faculty of Medicine, University of Coimbra, 3004-531 Coimbra, Portugal; 5 Centre for Innovative Biomedicine and Biotechnology (CIBB), University of Coimbra, 3000-075 Coimbra, Portugal; 6 Clinical Academic Center of Coimbra (CACC), 3030-370 Coimbra, Portugal; 7 Faculty of Dental Medicine, U. Porto, 4200-393 Porto, Portugal; 8 LAQV/REQUIMTE, U. Porto, 4160-007 Porto, Portugal

**Keywords:** orthodontics, cleft palate, cleft lip, survey

## Abstract

(1) Background: Orthodontists have an important role in cleft care. Over the two decades since the Eurocleft studies, a significant improvement in healthcare systems has been achieved but there has been no critical assessment regarding the establishment of proposed standard protocols. This study aimed to describe the current provider characteristics, orthodontic appliances, services offered, orthodontic complications, and cost analysis of cleft treatment in Europe. (2) Methods: A cross-sectional 22-question online survey, accessible from January 2021 to July 2021, was sent to 214 practitioners, pertaining to provider characteristics, orthodontic appliances, services offered, orthodontic complications, and cost analysis. Descriptive statistics were calculated for each question. Fisher’s exact test was used to assess the association between categorical variables. (3) Results: A total of 79 responses from 23 European countries completed the survey (response rate = 37%), with 69 surveys being assessed after the exclusion of incomplete surveys. Rapid maxillary expansion was the preferred expansion protocol (45%). Distraction osteogenesis was the most reported alternative treatment to secondary bone grafts (19%), with private practitioners being less likely to perform these treatments (Fisher’s exact test, *p* = 0.001). Orthodontic services offered were, however, rather similar in the various locations of provision (hospital and/or university, private). Compromised oral hygiene (77%) was the most reported orthodontic complication. The National Health Services support the majority of cleft orthodontic care (67%) in Europe. (4) Conclusion: An apparent improvement in orthodontic healthcare provision has been achieved within Europe in the last two decades, but there are several discrepancies, namely regarding treatment timing and the appliances offered.

## 1. Introduction

Cleft lip and palate (CLP) is one of the most frequent craniofacial malformations, affecting about 14.5:10,000 births between 2011 and 2018 in 26 European countries [1]. The management of patients with CLP requires an interdisciplinary team of specialists to achieve normal speech, hearing, and occlusion with a normal facial appearance and psychological well-being [2]. 

Several organisations have attempted to provide recommendations to reduce the burden in patients with CLP, notably the World Health Organization and the American Cleft Palate-Craniofacial Association (ACPA) [3,4,5]. In 2015, the latter association defined minimum standards for the inclusion in cleft palate teams, namely the fulfilment of the 8 basic criteria and 30 of the 35 additional criteria defined by the ACPA. The following criteria are relevant: the team must include an active orthodontist, surgeon, and speech-language pathologist; the orthodontist must have treated at least ten patients with cleft lip and/or palate in the prior year to the team’s application; and an orthodontist capable of providing orthodontic treatment as part of orthognathic treatment [6]. 

Despite improvements in both aesthetics and function, the surgical protocols used can lead to abnormal craniofacial development, associated with the formation of postoperative scar tissue [7]. Moreover, dental anomalies are significantly more frequent in patients with CLP than in the general population, with studies reporting that about 94% of patients with CLP present at least one dental anomaly [8]. An orthodontist has an important role in the interdisciplinary team, because some characteristics of patients with CLP may lead to the development of malocclusions (e.g., anterior or posterior crossbites, open bite, skeletal Class III and crowding) [9,10]. However, the majority of studies regarding orthodontic treatment in patients with CLP presented differences in the treatment protocols, measurement of outcomes (e.g., cephalometric analysis), characteristics of retrieved data and differences between control groups [11,12]. 

The Eurocleft network, involving 30 countries and 201 centres, highlighted the differences in cleft teams and treatment protocols in European countries. In 2001, some of these countries did not yet have an interdisciplinary team for cleft care [13]. Furthermore, significant variations were reported between centres, namely a mean of 3.5 to 6 surgical procedures and a length of orthodontic treatment of 3.3–8.5 years, with a range of 49–94 orthodontic visits [14]. After these disappointing findings, the EUROCRAN project emerged in order to promote international collaboration and study genetic and environmental risk factors for CLP, as well as prevention measures and treatment approaches [15]. More recently, a European cleft and craniofacial initiative for equality in care network was also created to promote the sharing of research methods and knowledge of treatments [16]. 

In the 18 years since the Eurocleft studies, healthcare systems all over Europe have undergone significant improvements, but several discrepancies between provider characteristics, treatment protocol or appliances offered, and financial support still persist [5,17]. These data might reveal that the recommendations of the aforementioned European projects were not consistently implemented. Standardised care will help to distribute available resources in a sustainable fashion and reduce the overall burden and cost of treatment. Thus, the aim of this study was to instigate a critical appraisal of orthodontic treatment in patients with CLP including provider characteristics, orthodontic appliances, services offered, orthodontic complications and cost analysis.

## 2. Materials and Methods

This study was approved by the Ethics Committee of the Faculty of Medicine of University of Coimbra (reference CE-071/2020). The study was handled in accordance with the Declaration of Helsinki. A cross-sectional 22-question survey was created to investigate several factors related to the provision of orthodontic cleft care, namely characteristics of care providers, size of treatment centres, orthodontics appliances offered, orthodontic complications, and financial support. For each question, participants had to select their answers from a list provided (Appendix A). 

The survey was developed using the Google documents platform with a unique URL, which allowed it to be accessed anywhere and at any time in the world. 

An email was sent with the survey link through the collection of contacts obtained through cleft and orthodontic association membership lists, namely the European Federation of Orthodontic Specialist Associations, European Orthodontic Society, European Cleft Organisation, and Orphanet. Unresponsive contacts were reminded by email at 2, 4, and 8 weeks after initially sending the link. The online survey was accessible from January 2021 until July 2021. The data were automatically stored with a unique study ID, ensuring the confidentiality of all data. An automated method generated the numeric data into an Excel spreadsheet (Microsoft Corporation, Redmond, WA, USA). Double entries and answers with disparities were manually rejected in order to create a final data set. 

The final data were extracted to the Statistical Package for the Social Sciences, version 24.0 for Windows (SPSS Inc., Chicago, IL, USA). Descriptive statistics were calculated for each question. Fisher’s exact test was used to assess the association between categorical variables. The chi-square goodness-of-fit test was used to evaluate the frequency distribution in multiple-choice questions, assuming a uniform distribution in the null hypothesis. The significance level was sent at *p* < 0.05.

## 3. Results

**Figure 1:**
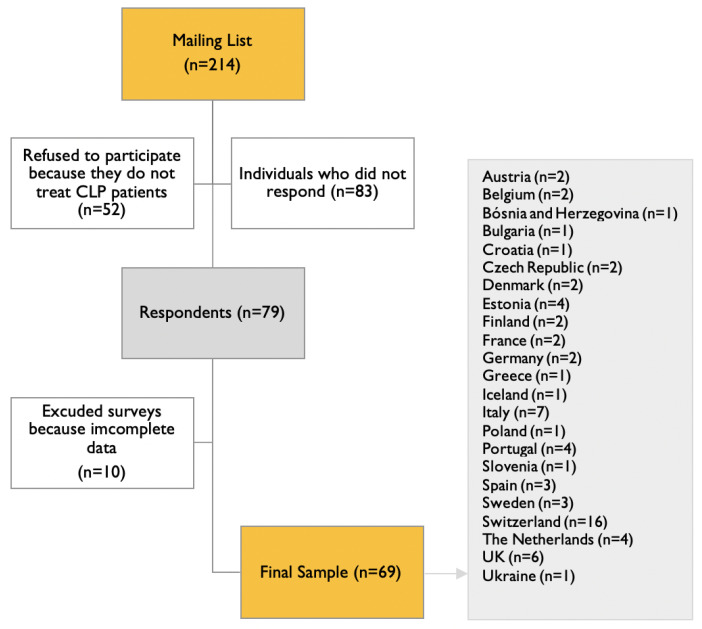
Flow chart of acquisition of final sample.

A total of 79 responses were obtained from 214 individuals contacted (response rate 36.9%). Of these, 10 responses were excluded due to the participants not having completed the entirety of the survey. The final sample thus consisted of 69 responses from 23 European countries (Figure 1). 

The data from the surveys revealed that in most cleft teams, orthodontists are present in the multidisciplinary team meetings more than 75% of the time, but the time dedicated to CLP care by these orthodontists is less than 26% (Table 1). No statistically significant association (Fisher’s exact test, *p* = 0.919) was found between the percentage of time that orthodontists are present in the multidisciplinary team meetings and the environment in which the centre or office was integrated (private practice or hospital and/or university environment).

The majority of the respondents (70.9%) reported offering presurgical orthopaedics, with bilateral CLP being the most common phenotype in which these appliances were used (Table 1).

Maxillary expansion is commonly used in the treatment protocol in patients with CLP. Of all maxillary expansion protocols, rapid maxillary expansion was preferred (44.9%), but no statistically significant differences were found (Fisher’s exact test, *p* = 0.886) between the different types of expansion. Furthermore, no association was found between the percentage of time a practitioner spent treating patients with CLP and the protocol or type of orthodontic appliance used for maxillary expansion (Table 2). Various types of orthodontic expansion appliances were used.

Distraction osteogenesis was the most reported alternative treatment to secondary alveolar bone grafts (18.8%) (Table 1). Additionally, we found that those in private practice were less likely to perform alternative treatments (Fisher’s exact test, *p* = 0.001). Regarding the timing of initiating tooth movement into the newly grafted area, the results were not homogeneous, with 3- and 6-month post-grafting being the most reported (Table 1).

**Figure 2:**
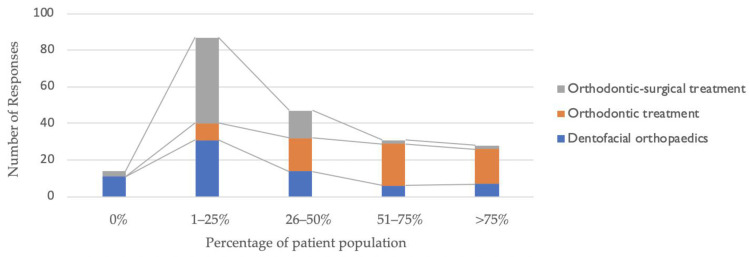
Distribution of patient population by type of orthodontic treatment.

The provision of orthodontic services was similar in the four types of practice locations (private practice, hospital, university, hospital, and university environment): dentofacial orthopaedics (Fisher’s exact test, *p* = 0.527); orthodontic treatment only (*p* = 0.587); and orthodontic surgical treatment (*p* = 0.110). Figure 2 describes the distribution of the patient population by the type of orthodontic treatment carried out. 

Orthodontic procedures were reported to have few complications, with compromised oral hygiene (77%) being the most reported (Table 3). Regarding pre-surgical orthopaedics, complications were mainly skin sores from the tape (28%).

Regarding cost analysis, we found that the National Health Services supported the majority of cleft orthodontic care (67%), and most patients reported to their medical team that they were satisfied with the services provided (74%) (Table 4). More than half of the respondents (61%) reported their patients having more than six appointments per year and the majority living less than 101 kilometres away (76.8%).

## 4. Discussion

In this study, we attempted to summarise the current provision of CLP orthodontic treatment in European countries. We also conducted a critical assessment of the implementation of the recommendations defined by the Eurocleft studies and European projects. 

Several descriptions of orthodontists’ role in the treatment of patients with CLP have been published, but significant variations have been reported concerning the availability of the orthodontist and the type of services offered [5,14]. This survey found that few orthodontists (10%) dedicated more than 75% of their time to CLP treatment. Two reasons may account for this. State-supported institutions (universities and/or hospitals) may have more difficulties in hiring full-time orthodontists due to financial restrictions. Additionally, 26.1% of respondents practiced in a private environment, which may also have contributed to the underestimation of this percentage, because, in this environment, it may be less common to have contact with patients with clefts. We found an improvement since the Eurocleft study, where the orthodontist was not recognised as one of the main specialties involved in cleft surgery, but presurgical orthopaedics was used by 65% of the teams [13]. More recently, in 2014, Scott et al. investigated the current provision of cleft services in the United Kingdom and found that primary cleft surgery and orthodontics were the only medical specialties represented in all the teams [17]. Additionally, Khavanin et al.’s study, published in 2019, showed that the majority of orthodontists integrated in cleft teams in the United States of America were private practice volunteers (48.6%), and only 17.1% devoted a majority of their practice (75% to 100%) to cleft care [5]. Although there is a discrepancy in the location of provision between Europe and the USA, the time devoted to CLP treatment is similar.

The timing and sequencing of orthodontic care are usually performed according to age and dental development. Regarding orthodontic appliances, this study was planned to examine the current provision of presurgical orthopaedics, maxillary expansion, and other appliances (e.g., speech bulbs). Despite the potential stated advantages in the literature of presurgical orthopaedics (e.g., cleft reduction, which promotes less lip tension and postoperatively benefits wound healing), only half of the respondents performed nasoalveolar moulding, which may be associated with the lack of evidence on long-term outcomes [18,19,20]. The role of maxillary expansion is already recognised as part of the treatment protocol for patients with CLP, with some goals such as the correction of the transverse discrepancy, establishment of the maxillary arch form, and availability of space for secondary bone grafts [21]. We found that respondents preferred the rapid maxillary expansion protocol. Previous studies suggested slow instead of rapid maxillary expansion based on the following assumptions: (a) patients with CLP have a disturbed, irregular, or absent palatal suture system, which increases the response to orthopaedic forces; (b) rapid maxillary expansion has some disadvantages such as requiring patient cooperation in activation, the creation of an open bite, micro-trauma of the temporomandibular joint, root resorption, tissue impingement, and pain; (c) slow maxillary expansion produces less tissue resistance around the circum-maxillary structures, which improves bone formation in the intermaxillary suture [22]. However, recent studies showed that both expansion protocols have similar effects [23]. The choice for the design of the expander may depend on factors such as practitioner preference or the localisation of the constricted region of the maxilla. Both hyrax and quad-helix expanders can be used when anterior and posterior maxillary expansion is needed, which may explain why these appliances were the most mentioned by the respondents [24]. Despite the potential benefit of a speech bulb in reducing hypernasality when velopharyngeal insufficiency is present, only a minority of responders (27.5%) offered this appliance [25]. This may have been due to the improvement in surgical protocols and the reduction in the necessity and indication of these appliances. 

Subsequently, secondary bone grafting may be required, which demands presurgical orthodontics that normally includes incisor alignment and control of the canine eruption [21]. The timing of the bone graft is dependent on dental development; usually, it is performed when the canine has one-half to two-thirds root formed, which typically occurs between 9 and 11 years [26]. After the secondary alveolar bone graft, orthodontic treatment should be restarted in order to move the teeth adjacent to the cleft area into the newly grafted bone, thus improving the consolidation of the alveolar bone and height of the crest. The timing to move the teeth into the grafted bone is not well-established in the literature; the most reported timing in the present survey was three to six months post-grafting [27]. Finally, if craniofacial development favourably occurs, fixed orthodontic appliances continue to re-establish facial aesthetics and proper function. However, if a skeletal discrepancy develops, the orthodontist should consider the previous surgical history, severity of skeletal discrepancy, and cessation of craniofacial growth to plan the orthodontic treatment [28]. Patients with more severe discrepancies may need a combined surgical and orthodontics treatment to obtain a normal occlusion and good support for the nose and upper lip [29].

This survey showed that the offer of common orthodontic services (removable and fixed appliances) is similar in various locations of the provision (private practice, hospital, university, hospital, and university environment), but the offer of less common treatment approaches may vary (less likely in private practice). These findings can be partially explained by several reasons: universities or hospital centres have several specialties on-site; contact with high volumes of patients allows more training in cleft management, and universities or hospital centres enjoy considerably higher rates of treatments covered by the National Health Service than those covered by private centres, allowing patients to access more expensive treatments, namely distraction osteogenesis. 

The orthodontic burden of care usually is higher in patients with CLP than those without this condition. Few complications regarding orthodontic treatment were reported (90% of respondents reported less than 26% complications), and the most reported complication was compromised oral hygiene. Patients with CLP may have more difficulty in performing adequate oral hygiene practices due to factors related to the higher incidence of supernumerary teeth, presence of malocclusions, presence of viscous nasal fluid that may accelerate the adherence of plaque, and healing tissue after surgical procedures that may make oral hygiene more challenging [30]. The duration of treatment also contributes to the orthodontic burden because patients with CLP may require more appointments (44 vs. 18 appointments in healthy patients) due to the complexity of the treatment [31,32]. 

The majority of respondents mentioned that the National Health Service supports cleft orthodontic care (67%). A distinct finding was obtained by Khavanin et al., showing that only a minority of orthodontic treatment was paid for by state-funded program in the USA [5]. This reveals the efforts of many countries within Europe to provide equal access to orthodontic treatment, respecting the third goal of the 2030 Agenda (ensure healthy lives and promote well-being for all at all ages). Moreover, most respondents reported that patients were satisfied with the orthodontic treatment offered (74%), which may be due to the variety of services available as well as the proximity of the centre or office of provision (most patients live less than 101 kilometres, 76.8%). 

A limitation of the present study was that the data may be biased because respondents provided their own subjective interpretations and perceptions. However, this survey represented a variety of practices, which may have minimised this bias. The second limitation of this study is the low response rate (37%). However, this rate is similar to those of other studies, namely the Eurocleft study (response rate = 40%) and the ACPA study about orthodontic care (response rate = 49.1%). Despite these findings needing to be interpreted with caution, they might be considered reflective of the current orthodontic practice in patients with CLP in Europe.

A significant improvement in orthodontic treatment provision was observed in Europe, but evidence in cleft treatment is still sparse because the results of current studies are heterogeneous due to several factors such as heterogeneous samples, different treatment protocols, and inadequate follow-up. Mossey et al. suggested the establishment of prospective registries to accelerate collaborative monitoring and critical appraisal (phase I trials) [33]. This would allow practice guidelines based on evidence to be established, improving surgical and orthodontic outcomes and reducing the burden of care, thus resulting in a reduced overall cost to the patient and society.

## 5. Conclusions

Europe has undergone an apparent improvement in orthodontic care over the past 18 years, but several discrepancies still exist, namely in the treatment timing protocol and appliances offered. Further research should be focused on the role of the orthodontic services in the outcome of cleft treatment.

## Figures and Tables

**Table 1:** Survey results on orthodontic care.

Question	Response	*p*-Value
Please select where your centre or office is integrated:		
Hospital environment	17.4%	0.401
University environment	24.6%	
Private practice	26.1%	
University hospital environment	31.9%	
What percentage of the time is an orthodontist present at your multidisciplinary clinic?		
0–25%	21.7%	<0.001
26–50%	13.0%	
51–75%	8.7%	
76–100%	56.5%	
What percentage of your orthodontist’s practice is devoted to the care of CLP patients?		
0–25%	57%	<0.001
26–50%	16%	
51–75%	17%	
76–100%	10%	
How often do you perform pre-surgical orthopaedics on CLP patients in your clinical practice?		
Always	18.8%	0.459
Often	30.4%	
Sometimes	21.7%	
Never	29.0%	
In which cleft phenotypes do you perform pre-surgical orthopaedics?		
Cleft lip	17.4%	NA
Cleft palate	29.0%	
Unilateral cleft lip and palate	56.5%	
Bilateral cleft lip and palate	62.3%	
Pierre Robin sequence	36.2%	
Other	13.0%	
In which cleft phenotypes do you perform nasoalveolar moulding?		
For patients with unilateral cleft lip and palate	21.7%	NA
For patients with bilateral cleft lip and palate	21.7%	
For all complete cleft lip and palate cases	18.8%	
Never	55.1%	
What is the usual maxillary expansion protocol that your team uses in CLP patients?		
Rapid maxillary expansion	44.9%	NA
Semi-rapid maxillary expansion	23.2%	
Slow maxillary expansion	39.1%	
Alt-RAMEC	8.7%	
What appliance do you normally use for maxillary expansion? (Choose up to 3 options)		
Removable expansion plate	23.2%	NA
Quad helix	53.6%	
Hyrax/Haas	55.1%	
W-Arch	8.7%	
NiTi expander	2.9%	
Spring Jet	14.5%	
Other tooth-borne appliance	2.9%	
Bonded expansion plate	15.9%	
Tooth-tissue-borne appliance	23.2%	
Bone-borne appliance	53.6%	
In CLP cases with a missing maxillary lateral incisor, which treatment approach do you prefer?		
Substitute the maxillary canines as the lateral incisor	27.5%	<0.001
Prosthetic replacement	4.3%	
No preference. The treatment approach depends on the clinical case	68.1%	
Other	27.5%	
After the alveolar graft, how long do you wait until you start moving teeth to the newly grafted area?		
1 month	7.2%	<0.001
2 months	14.5%	
3 months	34.8%	
4 months	2.9%	
5 months	1.4%	
6 months	34.8%	
Other	4.3%	
What alternative treatments do you use instead of secondary bone grafts?		
Distraction osteogenesis	18.8%	<0.001
Tongue flap and secondary bone graft	2.9%	
BMP-2	5.8%	
None	58.0%	
Other	17.4%	
Do you make other appliances (speech bulbs, etc.) for CLP patients?		
Yes	27.5%	<0.001
No	72.5%	

NA—not applicable (nonmutually exclusive choices).

**Table 2:** Correlation between CLP practice time and the protocol or type of orthodontic appliance.

Question	Answer Options	Fisher’s Exact Test
Protocol expansion	Rapid maxillary expansion	*p* = 0.361
	Semi-rapid maxillary expansion	*p* = 0.212
	Slow maxillary expansion	*p* = 0.406
	Alt-RAMEC	*p* = 0.244
Expansion appliances	Removable expansion plate	*p* = 0.244
	Quad helix	*p* = 0.313
	Hyrax	*p* = 0.105
	Haas	*p* = 1.000
	NiTi expander	*p* = 0.684
	Other tooth-borne appliance	*p* = 0.413
	Tooth-tissue-borne appliance	*p* = 1.000
	Bone-borne appliance	*p* = 0.133

**Table 3:** Complications of orthodontic procedures.

Question	Response
What percentage of CLP patients have complications during orthodontic or surgical treatment?	
0–25%	90%
26–50%	9%
51–75%	1%
76–100%	0%
What is the most frequent complication of pre-surgical orthopaedics?	
Interference with growth	12%
Delaying surgery	9%
Occlusion of the airway	1%
Skin sores from the tape	28%
Ulceration under a plate	16%
Risk of infection under a plate	3%
Feeding problems	6%
Other	41%
What is the most frequent complication of orthodontics treatment?	
Expansion relapse	52%
Ulceration	9%
Compromised oral hygiene	77%
Phonetic problems	13%
Mastication problems	7%
Dysphagia	0%
Other	4%

**Table 4:** Cost analysis.

Question	Response
How far from your centre or office are the majority of your CLP patients?	
0–50 km	46.4%
51–100 km	30.4%
101–150 km	13.0%
>150 km	10.1%
How many appointments do CLP patients have at your centre or office each year?	
0–3	23%
4–6	16%
7–12	49%
>12	12%
How do CLP patients typically pay for orthodontic services?	
National Health Service	67%
Out-of-pocket expenses (self)	10%
Insurances	7%
National Health Service and self-paid	12%
Insurances and out-of-pocket expenses (self)	4%
Are your patients satisfied with their current level of access to orthodontic services?	
Dissatisfied	1%
Neutral	10%
Satisfied	74%
Not sure	14%

## Data Availability

The data presented in this study are available on request from the corresponding author.

## Appendix A. 22-Question Survey

**Question**	**Answer Options** **(If Applicable)**
In what country do you practise?	
How many years has your centre/office collaborated in CLP treatment?	
Please select where your centre/office is integrated:	Hospital environment
	University environment
	Private practice
	University hospital environment
What percentage of the time is an orthodontist present at your multidisciplinary clinic?	0–25% 26–50% 51–75% 76–100%
What percentage of your orthodontist’s practice is devoted to the care of CLP patients?	0–25%; 26–50%; 51–75%; 76–100%.
Please tick the approximate percentage of your patient population in each category: 1. Dentofacial orthopaedics 2. Orthodontic treatment 3. Orthodontic-surgical treatment	0–25% 26–50% 51–75% 76–100%
How often do you perform presurgical orthopaedics on CLP patients in your clinical practice?	Always
	Often
	Sometimes
	Never
In which cleft phenotypes do you perform presurgical orthopaedics?	Cleft lip
	Cleft palate
	Unilateral cleft lip and palate
	Bilateral cleft lip and palate
	Pierre Robin sequence
	Other
In which cleft phenotypes do you perform nasoalveolar moulding?	For patients with unilateral cleft lip and palate For patients with bilateral cleft lip and palate For all complete cleft lip and palate cases Never
What is the usual maxillary expansion protocol that your team uses in CLP patients?	Rapid maxillary expansion Semirapid maxillary expansion Slow maxillary expansion Alt-RAMEC
What appliance do you normally use for maxillary expansion? (Choose up to 3 options)	Removable expansion plate Quad helix Hyrax/Haas W-Arch NiTi expander Spring Jet Other tooth-borne appliance Bonded expansion plate; Tooth-tissue-borne appliance Bone-borne appliance
In CLP cases with a missing maxillary lateral incisor, which treatment approach do you prefer?	Substitute the maxillary canines as the lateral incisor Prosthetic replacement No preference, treatment approach depends on the clinical case Other
After the alveolar graft, how long do you wait until you start moving teeth to the newly grafted area?	1 month 2 months 3 months 4 months 5 months 6 months Other
What alternative treatments do you use instead of secondary bone grafts?	Distraction osteogenesis Tongue flap and secondary bone graft BMP-2 None Other
Do you make other appliances (speech bulbs, etc.) for CLP patients?	Yes No
What percentage of CLP patients have complications during orthodontic or surgical treatment?	0–25%
	26–50%
	51–75%
	76–100%
What is the most frequent complication of presurgical orthopaedics?	Interference with growth Delaying surgery Occlusion of the airway Skin sores from the tape Ulceration under a plate Risk of infection under a plate Feeding problems Other
What is the most frequent complication of orthodontics treatment?	Expansion relapse Ulceration Compromised oral hygiene Phonetic problems Mastication problems Dysphagia Other
How far from your centre/office are the majority of your CLP patients?	0–50 km 51–100 km 101–150 km >150 km
How many appointments do CLP patients have at your centre/office each year?	0–3 4–6 7–12 >12
How do CLP patients typically pay for orthodontic services?	National Health Service Out-of-pocket expenses (self) Insurances National Health Service and self-pay; Insurances and out-of-pocket expenses (self)
Are your patients satisfied with their current level of access to orthodontic services?	Dissatisfied Neutral Satisfied Not sure
